# Network-Driven Insights into Plant Immunity: Integrating Transcriptomic and Proteomic Approaches in Plant–Pathogen Interactions

**DOI:** 10.3390/ijms27031242

**Published:** 2026-01-26

**Authors:** Yujie Lv, Guoqiang Fan

**Affiliations:** 1 Institute of Paulownia, Henan Agricultural University, Zhengzhou 450002, China; sgdyxe@163.com; 2 College of Forestry, Henan Agricultural University, Zhengzhou 450002, China

**Keywords:** disease resistance, gene regulatory networks, multi-omics, plant immunity, proteomics, transcriptomics, systems biology

## Abstract

Plant immunity research is being reshaped by integrative multi-omics approaches that connect transcriptomic, proteomic, and interactomic data to build systems-level views of plant–pathogen interactions. This review outlines the scope and methodological landscape of these approaches, with particular emphasis on how transcriptomic and proteomic insights converge through network-based analyses to elucidate defense regulation. Transcriptomics captures infection-induced transcriptional reprogramming, while proteomics reveals protein abundance changes, post-translational modifications, and signaling dynamics essential for immune activation. Network-driven computational frameworks including iOmicsPASS, WGCNA, and DIABLO enable the identification of regulatory modules, hub genes, and concordant or discordant molecular patterns that structure plant defense responses. Interactomic techniques such as yeast two-hybrid screening and affinity purification–mass spectrometry further map host–pathogen protein–protein interactions, highlighting key immune nodes such as receptor-like kinases, R proteins, and effector-targeted complexes. Recent advances in machine learning and gene regulatory network modeling enhance the predictive interpretation of transcription–translation relationships, especially under combined or fluctuating stress conditions. By synthesizing these developments, this review clarifies how integrative multi-omics and network-based frameworks deepen understanding of the architecture and coordination of plant immune networks and support the identification of molecular targets for engineering durable pathogen resistance.

## 1. Introduction

Plant pathogen interactions lie at the heart of plant health, crop productivity, and ecosystem stability. In these molecular engagements, plants deploy immune surveillance systems while pathogens evolve virulence strategies, creating a dynamic arms race. Pathogens such as fungi, bacteria, viruses, nematodes, and oomycetes threaten global food security, with disease outbreaks responsible for yield losses as high as 20–30% in staple crops under certain conditions [1]. Factors including agricultural intensification, climate change, and pathogen evolution accelerate the urgency to unravel how plants perceive and integrate complex environmental signals into defense. Historically, plant immunity research adopted reductionist approaches: dissecting genes, proteins, or signaling pathways individually. Such work yielded foundational discoveries—pattern recognition receptors (PRRs), resistance (R) genes, and the dual-layer models of pattern-triggered immunity (PTI) and effector-triggered immunity (ETI) [2]. However, these approaches fall short in capturing the multi-dimensional complexity of immune regulation, especially under variable or overlapping stress conditions.

Plants perceive pathogens through a multilayered immune system that begins with the recognition of conserved microbial signatures. PRRs, such as receptor-like proteins (RLPs) and receptor-like kinases (RLKs), detect pathogen-associated molecular patterns (PAMPs) and initiate PAMP-triggered immunity (PTI) [3,4]. This early perception activates rapid signaling events such as reactive oxygen species (ROS) generation, calcium influx, mitogen-activated protein kinase (MAPK) cascades, and transcriptional induction of defense-related genes [5,6]. Similarly, intracellular nucleotide-binding leucine-rich repeat receptors (NLRs) recognize pathogen effectors and trigger effector-triggered immunity (ETI), a stronger and often more localized immune response [7,8]. Together, these molecular actors regulate downstream defense outputs including phytohormone signaling (jasmonic acid, salicylic acid, ethylene), antimicrobial metabolite biosynthesis, and cell wall reinforcement [9,10,11].

Understanding these interconnected processes requires an integrated multi-omics perspective, as immune activation changes protein levels, transcript abundance, post-translational modifications, and metabolite fluxes simultaneously. Each omics layer alone captures only part of this dynamic defense system. Transcriptomics reveals large-scale changes in co-expression modules, gene expression, and immune-related regulatory signatures during plant–pathogen encounters [12]. However, mRNA abundance does not consistently predict protein levels or activation states. Proteomics complements transcriptomics by capturing changes in immune receptors, signaling proteins, and PTMs that modulate defense responses [13]. These layers interact closely during pathogen perception and response, making their integration essential for understanding how signals propagate from recognition to physiological output.

Integrating proteomics and transcriptomics within a multi-omics framework therefore provides a robust mechanistic foundation for dissecting plant–pathogen interactions. Such integration enables reconstruction of regulatory hierarchies, linking receptor activation to downstream protein networks and connecting expression signatures to functional immune responses [14,15,16]. A systems-level introduction is thus necessary to justify the review’s emphasis on multi-omics approaches that illuminate how plants perceive pathogens, coordinate signaling pathways, and mount effective defense responses.

The rise of high-throughput “omics” technologies has transformed this paradigm. Transcriptome profiling via RNA sequencing (RNA-seq) now enables detection of low-abundance transcripts, alternative splicing, non-coding RNAs, and subtle differential expression dynamics [17,18]. Proteomics driven by liquid chromatography-tandem mass spectrometry (LC-MS/MS) and quantitative strategies such as data-independent acquisition (DIA) and tandem mass tags (TMT) provides direct insight into protein abundance, post-translational modifications, and dynamic protein interactions [12,15]. Because mRNA and protein levels often diverge due to translational control, protein turnover, and post-translational modifications, proteomics and transcriptomics are complementary rather than redundant methods [19]. Integrating multiple omics layers marks a paradigm shift in plant systems biology. Multi-omics frameworks combine transcriptomic, proteomic, metabolomic, and epigenomic data to reconstruct regulatory networks that bridge genotype to phenotype [20]. In Arabidopsis thaliana and Oryza sativa, integrated transcriptome–proteome studies have revealed conserved defense modules and kinase cascades that would not emerge from single-layer analyses [15,21]. The addition of metabolomics and epigenomics further illuminates metabolic reprogramming and chromatin-mediated immune priming [22,23].

Central to this integrative approach is the concept of gene regulatory networks (GRNs). GRNs model the interactions among transcription factors, signaling proteins, non-coding RNAs, and target genes that orchestrate defense activation [24]. Computational inference tools such as GENIE3, ARACNe, and SCENIC have been applied to reconstruct GRNs from multi-omics datasets, highlighting key regulators of resistance or susceptibility [25,26]. Combining GRNs with time-series omics data enables dynamic modeling of immune responses and inference of causal regulatory links [27]. In this review, we examine how integrative multi-omics and GRN-based strategies have transformed our understanding of plant–pathogen interactions. We emphasize methodological innovations, representative case studies, and the fusion of transcriptomic and proteomic insights within network-level frameworks. We further extend discussion to multi-stress resilience, considering concurrent biotic and abiotic challenges (e.g., drought, salinity, temperature extremes). Finally, we highlight major integration bottlenecks and propose future directions toward precision systems biology in plant immunity.

## 2. Literature Search and Methodological Approach

This review employed a structured and transparent literature search strategy to ensure comprehensive coverage of recent advances proteomics-, in transcriptomics-, and network-based studies of plant–pathogen interactions. Peer-reviewed publications were identified through PubMed, Web of Science and Scopus, using combinations of keywords such as “plant–pathogen interactions”, “proteomics”, “transcriptomics”, “gene regulatory networks”, and “omics integration in plant immunity”. Although this manuscript focuses primarily on transcriptomic and proteomic insights, the term multi-omics is used to denote studies that integrate two or more molecular layers, particularly where proteogenomic or pathway-level connections are explored. The search was limited to publications from 2015 to 2025, with seminal earlier works retained for conceptual context. Of the 120 publications screened, 70 were selected based on clearly defined criteria: relevance, meaning a direct contribution to understanding plant defense or pathogen biology within an omics framework; methodological rigor, defined by the use of transparent workflows, validated omics platforms, and appropriate quality-control procedures; and conceptual contribution, interpreted as the study’s potential to advance integrative analysis, provide mechanistic insight, or introduce analytical innovation. Rather than excluding omics studies for being descriptive—common across high-throughput platforms—we included those in which descriptive data were interpreted within a mechanistic or network-level context. Purely observational studies lacking molecular data were excluded. Each selected study was evaluated for experimental design, omics technologies used, computational integration methods, and the biological significance of the findings. Analyzing these studies allowed for the identification of major trends, including advances in GRN inference, improved proteogenomic integration, and emerging tools like single-cell omics, AI-assisted data fusion, and spatial proteo-transcriptomics, which collectively highlight the rapidly evolving systems-level landscape of plant immunity research.

## 3. Advances in Transcriptomics and Proteomics in Plant–Pathogen and Multi-Stress Studies

Advances in transcriptomics have transformed the understanding of plant immune responses and stress adaptation by providing genome-wide and high-resolution insights into gene expression. High-throughput single-cell RNA-seq, RNA sequencing (RNA-seq), and spatial transcriptomics have enabled unprecedented resolution in quantifying transcriptional changes during pathogen infection and abiotic stress [27,28,29]. Transcriptomic profiling has revealed the transcriptional reprogramming that occurs upon recognition of PAMPs or effectors, shedding light on both effector-triggered immunity (ETI) or pattern-triggered immunity (PTI). Dual RNA-seq approaches, which simultaneously sequence host and pathogen transcripts, have further elucidated molecular cross-talk between plants and pathogens, as seen in rice–*Magnaporthe oryzae* and *Arabidopsis*–*Pseudomonas syringae* interactions (Table 1) [30,31].

Transcriptomic studies have highlighted how plants integrate signals from multiple stress pathways. For instance, *Arabidopsis* under combined drought and pathogen stress exhibits synergistic and antagonistic gene expression patterns involving ABA, JA and SA, signaling [7]. Similar regulatory modules have been identified in wheat and rice, where stress-responsive transcription factors such as WRKY, NAC, and MYB orchestrate cross-talk between biotic and abiotic stress networks [12,31]. Single-cell RNA-seq and spatial transcriptomics have refined these insights by identifying cell-type-specific defense modules. For example, maize root tissues under fungal attack revealed defense-associated pericycle cell clusters enriched in secondary metabolite biosynthesis genes [32]. Co-expression network analyses, such as weighted gene co-expression network analysis (WGCNA), have further pinpointed central regulators like SlWRKY33 and SlNAC1 in tomato that modulate both redox balance and defense priming under salt and pathogen stress [21]. Integrating transcriptomics with metabolomics has also helped link transcriptional regulation to metabolic adaptation during multi-stress responses [33].

**Table 1:** Case Studies of Transcriptomic Applications in Plant–Pathogen Interactions.

Plant–Pathogen System	Technology	Key Findings	Reference
*Arabidopsis–P. syringae*	Dual RNA-seq	Coordinated regulation of SA and JA signaling genes	[31]
*Rice–M. oryzae*	RNA-seq	Activation of WRKY and NAC transcription factors in resistance	[30]
*Wheat–Fusarium graminearum*	Time-series RNA-seq	Early upregulation of secondary metabolite biosynthetic genes	[12]
*Soybean–P. sojae*	scRNA-seq	Cell-specific defense gene induction and ROS signaling	[33]

### 3.1. Proteomic Insights into Multi-Stress Adaptation and Defense Mechanisms

**Figure 1:**
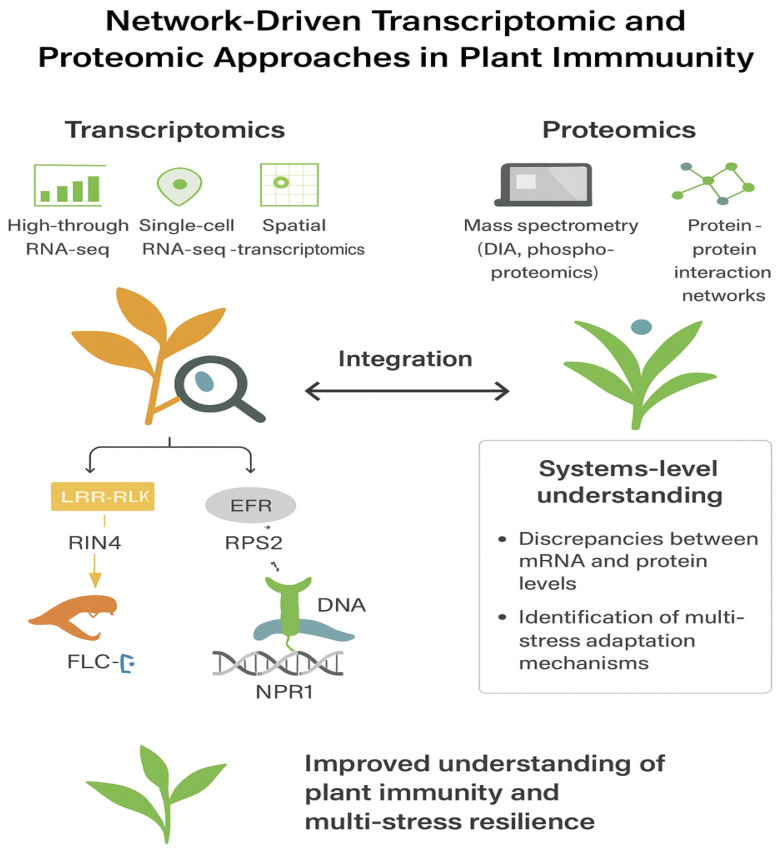
Transcriptomic workflows in plant–pathogen and stress studies. RNA-seq enables genome-wide expression profiling, identification of differentially expressed genes, and mapping of stress-responsive pathways and regulatory networks, providing mechanistic insights into plant immunity and multi-stress adaptation.

Proteomics complements transcriptomics by providing insight into protein modifications, post-transcriptional regulation, and dynamic signaling events. Comparative proteomic analyses have identified stress-responsive proteins involved in ROS scavenging, heat-shock responses, and pathogenesis-related (PR) protein accumulation [34,35]. In soybean, label-free quantitative proteomics during *Phytophthora sojae* infection revealed defense-associated proteins linked to phenylpropanoid metabolism and cell-wall reinforcement [36]. Similarly, phosphoproteomics in maize has uncovered phosphorylation-dependent activation of MAPK cascades and calcium signaling under combined drought and fungal stress [37] as illustrated in Figure 1.

Proteomics also emphasizes the importance of protein–protein interaction (PPI) networks in coordinating multi-stress responses. Interactome analyses in *Arabidopsis* have highlighted cross-network modules where ubiquitin ligases and kinases regulate proteostasis and immune signaling [38]. Recent advances, such as data-independent acquisition (DIA) mass spectrometry, have improved proteome coverage, enabling quantitative comparisons across developmental stages and stress conditions [25]. Integrating transcriptomic and proteomic data identifies key discrepancies between mRNA and protein levels due to translational control or protein degradation, offering critical insights into the mechanisms underlying plant adaptive plasticity [39].

### 3.2. Metabolomics Techniques and Integration with Transcriptomics/Proteomics

Metabolomics has become an indispensable component of plant multi-omics research, providing a direct biochemical readout of metabolic fluxes, defense activation, and pathogen-induced perturbations. Contemporary plant metabolomics relies heavily on gas chromatography–mass spectrometry (GC–MS), liquid chromatography–mass spectrometry (LC–MS/MS), and nuclear magnetic resonance (NMR) spectroscopy, each offering complementary coverage across volatile, semi-polar, and primary/secondary metabolites [40,41,42,43]. Untargeted profiling enables the discovery of novel biomarkers and pathway intermediates, whereas targeted metabolomics quantifies specific compounds such as oxylipins, phytoalexins, and pathogen-associated toxins. Integrating these datasets with transcriptomics and proteomics enhances pathway reconstruction by validating transcriptional predictions with downstream metabolite accumulation patterns [44]. For example, co-expression–metabolite correlation networks can identify regulatory hubs that coordinate defense metabolism, while joint multi-omics modeling can link transcription factor activity to metabolite flux, thereby refining gene regulatory network (GRN) predictions in plant–pathogen systems [45,46]. This integrative approach provides a more mechanistic understanding of disease resistance that transcends single-omic limitations.

### 3.3. In Vitro vs. InIn Planta Metabolomic Strategies and Future Perspectives

Metabolomic investigations in plant–pathogen research typically follows two complementary strategies: in vitro profiling and in planta analyses. In vitro approaches, like metabolite profiling of axenic co-culture systems, isolated pathogens, or elicitor-treated cell suspensions, enable high-resolution characterization of pathogen metabolites, enzymes, and toxins under controlled conditions [47]. However, in planta metabolomics provides a more ecologically realistic view by capturing spatially resolved changes in infected tissues, including redox shifts, localized defense compounds, and metabolic cross-talk between host and pathogen [48,49]. Recent advances, such as stable-isotope tracing, MALDI-MS imaging, and microdissected metabolomics, now allow for direct detection of pathogen toxin production within plant tissues and mapping of its downstream physiological consequences. Future integrative multi-omics studies should couple in planta metabolomics with transcriptomics, proteomics, and GRN inference frameworks to decode how toxins modulate host signaling networks, reprogram metabolic defenses, and contribute to susceptibility or resilience [50]. Such integrative strategies will be crucial for generating predictive models that link molecular regulation to disease outcomes, especially under concurrent biotic and abiotic stresses.

## 4. Gene Regulatory Networks (GRNs) and Systems-Level Integration

### 4.1. GRN Reconstruction and Network Dynamics

Gene regulatory networks (GRNs) give a description of the complex interactions between transcription factors, signaling molecules, and downstream target genes that determine plant responses to stress. Indeed. network-based modeling has become central in identifying key regulators of resilience and susceptibility [51,52]. Machine learning approaches such as Bayesian inference, random forest regression, and graph neural networks are currently increasingly being employed to infer regulatory interactions from omics datasets [53,54]. For instance, a study on integrated GRNs in rice revealed that WRKY and bZIP transcription factors form feedback loops linking defense and drought responses [38]. Moreover, dynamic network modeling in *Populus trichocarpa* also showed that network rewiring under heat stress enhances co-regulation of secondary metabolism and antioxidant defenses [33]. Therefore, time-series transcriptomics combined with chromatin accessibility data (ATAC-seq) has further contributed to the elucidation of temporal GRN dynamics, identifying early- and late-response modules [55]. Additionally, multi-layer network integration that incorporates miRNA, lncRNA, and epigenetic regulation has greatly enhanced predictive modeling of plant defense networks [30,56].

Recent advances in constructing gene regulatory networks (GRNs) that integrate multi-dimensional experimental approaches have revolutionized the study of plant responses to simultaneous biotic and abiotic stresses. Traditionally, GRN analyses focused on isolated stress factors such as drought, salinity, or pathogen attack, offering valuable insights but often failing to capture the complex, interactive nature of real-world stress environments [57]. Current research emphasizes integrative multi-omics frameworks combining transcriptomics, proteomics, metabolomics, and epigenomics to map dynamic and context-specific regulatory networks that mediate cross-tolerance mechanisms [58,59]. Through such integrative approaches, researchers have identified stress-responsive transcription factors (e.g., WRKY, DREB, and NAC families) that serve as regulatory hubs linking abiotic and biotic stress pathways, thereby coordinating responses through hormonal signaling, redox balance, and metabolic reprogramming [60,61].

Advancements in computational biology and network inference algorithms, including Bayesian modeling and machine learning, have further enhanced the resolution of GRNs, allowing for dynamic reconstruction of transcriptional interactions under multifactorial stress conditions [62,63]. For instance, integration of time-series transcriptomic data with chromatin accessibility profiles enables the identification of key regulators controlling stress-memory responses and transcriptional plasticity [10,64]. Such approaches reveal how plants integrate diverse stress cues through interconnected signaling cascades involving ABA, JA, and ROS, which collectively modulate defense prioritization and resource allocation [62,65]. Moreover, multi-stress GRNs are increasingly being applied to translational crop improvement, guiding CRISPR-based network re-engineering to enhance resilience under combined heat, drought, and pathogen pressures [66,67]. Collectively, these developments signify a major shift toward holistic modeling of plant stress adaptation. By integrating experimental and computational approaches, multi-stress GRNs provide a mechanistic framework that links molecular regulation to physiological resilience. This systems-level methodology represents the current frontier in plant stress biology, enabling predictive and design-oriented applications in climate-smart agriculture and sustainable ecosystem management [68].

### 4.2. Multi-Omics Integration Frameworks

The construction of gene regulatory networks (GRNs) under multi-stress conditions has marked a significant advance in plant stress biology, offering a paradigm shift from traditional single-stress analyses to systems-level integration of complex environmental interactions. Traditional studies that isolate abiotic (e.g., drought, salinity, heat) or biotic (e.g., pathogen, pest) stress responses have provided valuable mechanistic insights, yet have often failed to account for the non-linear, synergistic, and antagonistic effects that occur under simultaneous stress exposures [65]. Multi-stress GRNs integrate multi-omics data transcriptomics, proteomics, metabolomics, and epigenomics to reveal emergent properties that cannot be inferred from single-factor experiments alone [26,59]. Such integrative approaches allow for the identification of shared transcriptional hubs and signaling nodes that mediate cross-tolerance, including transcription factors such as DREB, NAC, WRKY, and MYB families, which coordinate overlapping stress pathways [59,69]. Moreover, systems-level modeling of multi-stress responses enables the mapping of regulatory motifs and feedback loops that underpin resilience, offering predictive frameworks for adaptive plasticity across genotypes and environments [58].

Dynamic GRNs incorporating temporal and spatial data further enhance our understanding of how plants prioritize resource allocation under competing stress demands [57]. For example, machine learning and Bayesian inference models are now employed to infer causal interactions from time-series transcriptomic data, revealing phase-specific regulators and signal integration nodes [62,63]. Multi-stress GRNs also expose cross-talk between hormonal and redox signaling pathways—particularly involving abscisic acid (ABA), salicylic acid (SA), and reactive oxygen species (ROS)—that mediate trade-offs between growth and defense [60]. These findings align with emerging network-theoretical views that conceptualize stress adaptation as a property of distributed regulatory architecture, where robustness arises from modular connectivity and redundancy among stress-responsive subnetworks [67]. Beyond mechanistic insights, the application of multi-stress GRNs has practical implications in synthetic biology and crop improvement, facilitating the rational design of resilient phenotypes through network-guided gene editing and transcriptional reprogramming [56,67].

Integrating GRNs under multi-stress conditions therefore extends beyond descriptive analyses it provides a quantitative and predictive framework linking molecular regulation to organismal performance and ecological fitness. By contextualizing transcriptional responses within multi-dimensional environmental data, these networks enable simulation of adaptive responses under realistic climate scenarios, offering pathways for breeding crops with enhanced multi-stress tolerance [68,69]. Furthermore, emerging computational pipelines now allow for the integration of spatiotemporal omics data with phenomics and environmental metadata, resulting in a new generation of dynamic, context-aware GRNs [53]. Addressing redundancy in the current literature thus requires moving beyond static network representations toward dynamic, experimentally validated models that incorporate feedback, stochasticity, and evolutionary constraints. In this context, developing visually and conceptually rich figures that depict network topology, regulatory motifs, and stress signal convergence will better communicate the conceptual depth of multi-stress GRN frameworks. Consequently, constructing multi-stress GRNs not only enhances mechanistic understanding but also bridges molecular insights with translational outcomes in sustainable agriculture and ecosystem resilience [66,68]. This frontier in stress biology underscores the power of integrative, data-driven modeling to uncover the coordinated orchestration of plant responses to the multifaceted pressures of a changing environment.

Integrating transcriptomics, proteomics, metabolomics, and epigenomics has further enabled a holistic view of plant–pathogen interactions and stress adaptation [70]. Data fusion approaches such as canonical correlation analysis, partial least squares regression, and multi-layer network alignment contributes to the facilitation of cross-omics interpretation [66,67]. In *Arabidopsis* studies, integrative analysis that combines RNA-seq, proteomics, and metabolomics data under cold stress has identified central regulators in flavonoid biosynthesis and membrane remodeling [59]. Similarly, a study that focused on systems integration in rice highlighted the cross-regulation of nitrogen metabolism and pathogen defense via transcription factor OsNAC6 [12]. Furthermore, the emerging field of predictive multi-omics modeling is integrating AI and network inference to simulate plant responses under combined stresses [58]. In such models, potential genetic targets for crop improvement using CRISPR-based editing of network hubs are being identified [60].

### 4.3. Applications and Translational Perspectives

Integrative gene regulatory network (GRN) and multi-omics approaches are transforming the translation of molecular insights into tangible agricultural innovations. These integrative strategies enable the systematic identification of transcriptional regulators, signaling nodes, and metabolic pathways that underpin plant adaptation to complex environmental stresses. By leveraging transcriptomic, proteomic, metabolomic, and epigenomic datasets, researchers can construct predictive models of stress response networks that guide precision breeding and genome editing programs [71]. Network biomarkers derived from such analyses serve as molecular indicators for selecting elite genotypes, facilitating marker-assisted and genomic selection in breeding pipelines [67]. Furthermore, the integration of omics with field-based phenomics and environmental data allows for the dynamic assessment of genotype–environment interactions, improving the prediction accuracy of stress resilience traits under variable field conditions. This translational framework bridges the gap between laboratory discoveries and field performance, accelerating the deployment of stress-tolerant cultivars [28]. Synthetic biology also offers powerful tools to reprogram regulatory circuits and engineer synthetic promoters or transcription factors that enhance plant robustness against drought, salinity, or pathogen pressure.

As climate change continues to amplify the frequency and intensity of combined abiotic and biotic stresses, integrating omics-driven insights into next-generation crop design is essential for achieving sustainable agricultural productivity and food security. Systems-guided crop improvement informed by GRN modeling will thus play a pivotal role in developing resilient crop varieties capable of thriving in rapidly changing environmental landscapes [58].

## 5. Proteomics in Plant–Pathogen Interactions

### 5.1. Proteomic Technologies

Proteomic technologies have evolved into indispensable tools that complement transcriptomic and metabolomic analyses by providing a direct assessment of protein abundance, post-translational modifications (PTMs), and dynamic signaling networks associated with plant defense responses. Unlike transcriptomics, which reflects potential gene expression, proteomics captures the actual functional state of the cell, revealing the biochemical processes that drive immunity. Many studies have applied advance high-resolution mass spectrometry (MS) platforms and quantitative techniques such as label-free quantification (LFQ), tandem mass tags (TMTs), and data-independent acquisition (DIA) to significantly expand proteome depth, sensitivity, and reproducibility, enabling precise quantification of thousands of proteins across diverse stress conditions [53,63]. Specialized branches of proteomics, including phosphoproteomics, ubiquitinomics, and acetylomics, have also been applied to further advance the understanding of how PTMs regulate plant immune signaling. Phosphoproteomics, for instance, reveals dynamic kinase and phosphatase activities central to pattern-triggered immunity (PTI), while ubiquitinomics uncovers mechanisms of protein turnover and receptor recycling that underpin effector-triggered immunity (ETI) [37,71]. These approaches provide key insights into the activation and fine-tuning of defense cascades.

Moreover, subcellular and organelle-specific proteomics have enabled the spatial mapping of defense-associated proteins, particularly those localized in chloroplasts, mitochondria, nuclei, and the apoplast regions crucial for energy metabolism and pathogen recognition [27,34]. Integrating proteomic datasets with transcriptomic and metabolomic profiles not only enhances the understanding of defense network architecture but also identifies potential molecular targets for engineering durable resistance in crops.

### 5.2. Applications and Case Studies of Proteomic Analyses in Plant–Pathogen Interactions

**Figure 2:**
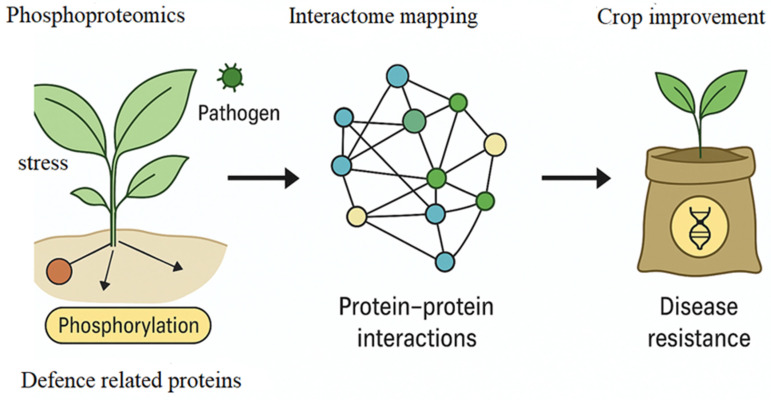
Proteomics-driven insights into plant immunity and crop improvement. Pathogen- or stress-induced phosphorylation events in plants modulate defense-related proteins, which can be systematically captured using phosphoproteomics. These phosphorylation changes are subsequently integrated into protein–protein interaction (PPI) networks through interactome mapping, enabling the identification of key regulatory hubs and signaling pathways underlying plant immune responses. Insights gained from this systems-level approach can be exploited to guide crop improvement strategies, ultimately contributing to enhanced disease resistance and stress tolerance.

The application of proteomic investigations has enabled the identification of stress-responsive proteins that mediate defense activation, detoxification, and energy reallocation. In soybean–*P. sojae*, this technology has shown that the interactions, phenylpropanoid metabolism enzymes and PR proteins are strongly upregulated [36]. Similarly, another study in *Oryza sativa* under M. oryzae infection, has shown that phosphorylation of MAPK components can enhance the signal amplification [35,43]. Moreover, the quantitative proteomics in wheat infected by *Puccinia triticina* uncovered proteins that are related to ROS detoxification and proteasome function [35]. Therefore, the uptake of such technological approaches has generated datasets that have continued to offer critical targets for breeding and biotechnology-based disease management. According to Figure 2, proteomic approaches, including phosphoproteomics and interactome mapping, reveal defense-related protein networks that enhance disease resistance and guide genetic improvement.

## 6. Integrating Transcriptomics and Proteomics in Plant–Pathogen Interactions

### 6.1. Correlation and Discordance Analysis

The integration of transcriptomic and proteomic datasets has become a cornerstone for understanding the multilayered regulatory mechanisms underlying plant–pathogen interactions. Although transcriptome profiling provides a snapshot of mRNA abundance, protein expression levels often diverge significantly, reflecting complex post-transcriptional, translational, and post-translational control mechanisms. Studies have consistently reported both concordant and discordant expression patterns between mRNAs and their corresponding proteins, with discrepancies attributed to mRNA stability, translation efficiency, and protein turnover [39]. For example, time-course analyses in *Arabidopsis thaliana* and rice (*Oryza sativa*) have shown that only about 40–60% of defense-related proteins exhibit strong correlations with transcript levels, underscoring the significance of translational regulation during immune responses [61].

These discordances often occur during rapid immune activation, where translation reprogramming or selective degradation ensures a timely defense response. For instance, under pathogen attack, specific ribosome-associated proteins enhance the translation of defense-related transcripts while suppressing growth-associated genes, reflecting a strategic reallocation of cellular resources. Moreover, protein stability is modulated through ubiquitination and proteasomal degradation, mechanisms that fine-tune receptor availability and signaling cascades during effector-triggered immunity (ETI). Therefore, analyzing mRNA–protein correlations provides vital clues about post-transcriptional control points that define the amplitude and duration of plant immune signaling.

### 6.2. Computational Integration Strategies

Integrating high-dimensional transcriptomic and proteomic data requires robust computational frameworks capable of disentangling complex relationships between transcriptional and translational layers. Several integrative bioinformatics tools such as iOmicsPASS, DIABLO (Data Integration Analysis for Biomarker discovery using Latent variable approaches for Omics studies), and Weighted Gene Co-expression Network Analysis (WGCNA) have been developed and applied to fuse multi-omics datasets and identify functionally coherent modules [59,67]. These methods allow for the detection of co-expressed gene–protein pairs, regulatory hubs, and subnetworks involved in stress responses. For instance, WGCNA constructs correlation networks that cluster genes and proteins into co-expression modules, which can then be linked to phenotypic traits such as pathogen resistance or stress tolerance. iOmicsPASS integrates multi-omics correlation structures to infer directional signaling relationships, while DIABLO applies multivariate dimension reduction to identify shared signatures across omic layers. In addition, machine learning algorithms—particularly Bayesian networks, support vector machines, and random forests—are increasingly used to model complex regulatory hierarchies and predict protein abundance from transcript data [7,9]. Emerging systems biology platforms also incorporate network inference with functional enrichment and subcellular localization data to uncover cross-talk between immune signaling and metabolic reprogramming. Such approaches not only enhance predictive accuracy but also facilitate the prioritization of key regulators for genetic engineering and genome editing.

### 6.3. Functional Insights from Integrated Analyses

The application of integrated transcriptome–proteome analyses has yielded transformative insights into plant immune regulation. By jointly analyzing both omics layers, researchers can capture the complete continuum from transcriptional induction to protein-level execution of defense responses. In Solanum lycopersicum, a combined transcriptomic–proteomic study identified WRKY33 as a central hub connecting jasmonate-mediated secondary metabolism with resistance to Botrytis cinerea, revealing new layers of transcription–translation coupling in defense signaling [21,24]. Similarly, integrative analyses in rice have unveiled that hormonal cross-talk, particularly between ABA, SA, and JA is regulated by coordinated transcriptional activation and selective protein translation during combined drought and pathogen stress [24].

Moreover, multi-omics studies have revealed that several metabolic enzymes, initially considered housekeeping, exhibit differential phosphorylation or ubiquitination during pathogen challenge, linking primary metabolism with immune reprogramming. For example, studies have shown that glycolytic enzymes and photosynthetic proteins are often downregulated at the protein level despite stable mRNA expression, suggesting targeted degradation to redirect energy toward defense compound biosynthesis. These integrative analyses have also uncovered novel translational regulators, such as RNA-binding proteins and small peptides, that modulate defense-associated mRNA stability and translation. Together, these findings highlight that a holistic understanding of plant–pathogen interactions requires not only mapping transcriptional changes but also deciphering their proteomic consequences. By uniting transcriptomics and proteomics, researchers can uncover mechanistic insights into plant immune plasticity, identify biomarkers of resistance, and accelerate the design of crops with durable and broad-spectrum disease resistance.

## 7. Deciphering Plant–Pathogen Interactomes

### 7.1. Host–Pathogen Protein–Protein Interactions (PPIs)

**Figure 3:**
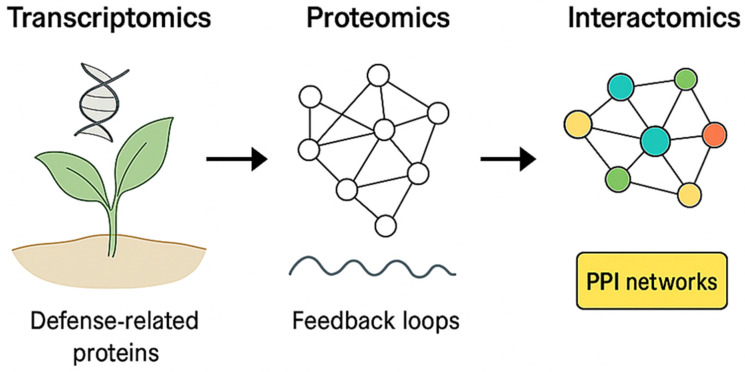
Conceptual overview of advancements in plant immunity research driven by multi-omics integration. Transcriptomic analyses capture stress- or pathogen-induced gene expression changes that give rise to defense-related proteins. Proteomic profiling reveals protein abundance dynamics and regulatory feedback loops operating downstream of transcriptional control. These layers are subsequently integrated through interactomics to construct protein–protein interaction (PPI) networks, enabling the identification of key signaling nodes and regulatory modules that coordinate plant immune responses.

Plant–pathogen interactions have been shown to be mediated by intricate PPI networks between host immune components and pathogen effectors [72]. High-throughput yeast two-hybrid (Y2H) and affinity purification–mass spectrometry (AP–MS) have been applied to map key nodes such as receptor-like kinases (RLKs) and R proteins [73]. Interactome studies have also revealed how pathogens target host hub proteins to suppress immunity, for example, *P. syringae* effectors has been shown to disrupt host kinases that are involved in defense signaling [22]. Indeed, Figure 3 depicts how transcriptomics, proteomics, and interactomics converge to reveal regulatory hierarchies, feedback loops, and PPI networks that control plant defense.

### 7.2. Multi-Omics Contributions to Interactome Mapping

Multi-omics data integration has continued to enrich interactome mapping by linking protein networks with transcriptomic co-expression and metabolite flux changes [51]. Specifically, phosphoproteomics combined with transcriptomics has revealed the role of calcium-dependent kinases in PTI amplification [35]. Moreover, the metabolite-informed interactomes have identified novel defense compounds and their regulatory enzymes, advancing the understanding of secondary metabolism during infection [39].

### 7.3. Multi-Layered Network Inference Approaches Uncovering Hormone–ROS–Defense Cross-Talk

**Figure 4:**
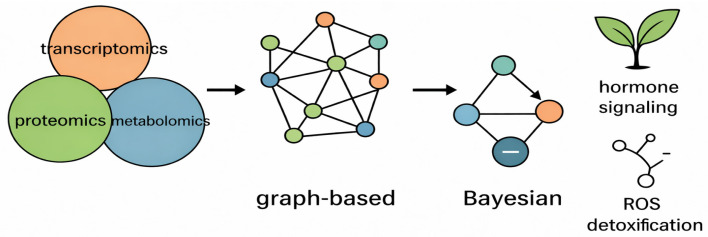
Integrative Multi-Layered Network Inference with Cross-Talk Between Hormone Signaling, ROS Detoxification, and Plant Defense Responses. Transcriptomic, proteomic, and metabolomic datasets are integrated to construct graph-based interaction networks that capture complex molecular relationships. Bayesian modeling is subsequently applied to infer causal links, regulatory directionality, and key control nodes within these networks. This integrative framework enables the identification of core pathways underlying plant stress adaptation, including hormone signaling and reactive oxygen species (ROS) detoxification.

Understanding how hormone signaling, ROS detoxification and immune responses are coordinated still requires methods able to fuse heterogeneous molecular layers (transcriptomes, proteomes, phosphoproteomes, metabolomes) and reveal both intra-layer and inter-layer interactions, as demonstrated in Figure 4. Multi-layered network inference combines graph-based topology analyses with probabilistic/casual models (e.g., Bayesian networks) and supervised multivariate fusion techniques to (a) detect co-regulated modules, (b) infer directionality or causal influence, and (c) prioritize regulatory hubs that mediate cross-talk among pathways [58,67]. Graph methods represent molecular entities as nodes and associations (correlation, partial correlation, physical interactions) as edges. They are widely used to detect co-expression or co-abundance modules that often correspond to hormone, redox, or defense functional groups.

Weighted correlation/module detection (WGCNA has been used to group genes/proteins into modules based on pairwise weighted correlations; module eigengenes can then be correlated with treatments or phenotypes to identify hormone- or ROS-linked modules [74]. Modules enriched for, e.g., ABA-responsive genes or antioxidant enzymes are candidate cross-talk nodes. Graph metrics (degree, betweenness, closeness) can also identify hub nodes transcription factors (TFs) or signaling kinases that bridge modules [75]. In plant stress studies, hubs often are TFs (WRKYs, NACs, MYC/MYB) with roles in both hormone signaling and defense activation. Additionally, studies have reported that network diffusion and random-walks propagate information from known hormone- or ROS-related seeds to discover neighboring subnetworks (useful when one has partial prior knowledge). Indeed, these emerging approaches embed node features (multi-omics measurements) into graph models, enabling powerful representation learning for cross-layer pattern detection (recent surveys; see [64] for single-cell omics applications). These methods are intuitive, scalable, and useful for discovering modules and hubs that are candidate mediators of cross-talk. Limitation: edges tend to be undirected and may conflate correlation with causation.

Bayesian networks and related probabilistic models aim to infer directed relationships (probabilistic dependencies) among variables, providing a route toward causal hypotheses. This approach has been applied to infer conditional dependence structures from steady-state data, flagging likely regulatory influences [76]. When TF expression (or phospho-states) probabilistically predicts downstream gene/protein abundance, the model suggests putative regulatory edges. The dynamic Bayesian networks (DBNs) and time-series models incorporating temporal omics (time-course transcriptomics + phosphoproteomics) allow for inference of time-lagged dependencies that are especially valuable for signaling cascades (hormone pulses → TF activation → antioxidant gene induction). DBNs can separate early hormone signaling events from later ROS detoxification responses.

Recent hybrid approaches have been applied to integrate prior knowledge (PPI, TF binding, hormone pathway maps) as priors into Bayesian models to increase robustness and reduce false positives [39], with the advantage of suggesting direction and can model uncertainty, which is critical when dissecting hormone-ROS-defense hierarchies. However, this approach still requires adequate sample size (and ideally time points) and can be computationally demanding for genome-scale networks.

To combine heterogeneous layers, several fusion strategies are commonly used, the latent variable and component models (e.g., DIABLO/mixOmics): DIABLO identifies latent components shared across omics layers that discriminate conditions (e.g., stressed vs. control) and selects correlated features across layers effective to extract multi-omics signatures linking hormone metabolites, TF transcripts, and defense proteins [58]. Knowledge-guided regression/network inference (e.g., KiMONo and related two-step methods): These methods have been applied to incorporate partial prior networks and use sparse regression/penalized models to infer inter-layer links, explicitly addressing missing data and heterogeneity [39]. Moreover, hybrid pipelines which is a graph detection (WGCNA) to find modules, followed by Bayesian/causal modeling between module eigengenes and targeted downstream traits, yields tractable and interpretable networks. These fusion methods have remained powerful in identifying cross-talk as they have made it possible to capture correlated signals across omics (e.g., hormone levels ↔ receptor phosphorylation ↔ TF activity ↔ antioxidant enzyme abundance), and to prioritize multi-omic biomarker panels that mediate cross-pathway integration [39,58]. Practically, multi-layer inference have revealed several recurrent motifs of cross-talk. Hormone signaling modules (ABA, JA, SA) have been shown to be linked to TFs (e.g., MYC2, WRKYs) that regulate antioxidant gene sets (peroxidases, superoxide dismutases), visible as inter-layer edges (transcript ↔ phospho-TF ↔ protein abundance). Phosphoproteomics that integrates with network inference highlights kinases that simultaneously modulate hormone receptor signaling and ROS-scavenging enzymes a mechanistic node of cross-talk.

## 8. Multi-Omics Case Studies in Specific Pathosystems

Multi-omics case studies have continued to provide practical insights into how integrative analyses translate into crop improvement strategies (Table 2).

## 9. Future Perspectives and Translational Pathways

### 9.1. Enhancing Multi-Stress, Spatio-Temporal Resolution

The emergence of technologies has increasingly supported multi-omics studies that have accurately captured spatio-temporal dynamics under combined biotic and abiotic stresses. Many studies have reported that single-cell RNA-seq and spatial transcriptomics are already revealing heterogeneous cell states during pathogen infection [12,13]. Therefore, applying these to multi-stress setups, e.g., drought + pathogen, heat + salinity + pathogen have allowed for the reconstruction of gene regulatory networks (GRNs) with cell-type specificity and temporal order. Indeed, the integration of time-series data from transcriptome, phospho-proteome, metabolome, and epigenome layers has remained critical in deciphering responses that are transient or delayed. For example, integrating transcriptome and phosphoproteome data in *Rhododendron chrysanthum* under UV-B and ABA treatments revealed faster phosphorylation changes preceding transcriptional reprogramming [62,68].

### 9.2. AI, Predictive Modeling, and Network Causality

Machine learning, deep learning, and Bayesian approaches have increasingly been applied in the study of crops not just for pattern detection, but for causal inference in GRNs. Generative models and latent variable approaches have so far assisted in predicting how signal transduction cascades operate under stress combinations. For instance, deep learning architectures tailored to omics data (e.g., graph neural networks) are have been shown to improve predictions of regulatory interactions, especially when trained on multi-layer and multi-stress datasets (PhytoCluster) [66]. Moreover, explainable AI (XAI) methods have been applied to interpret the molecular features (genes, proteins, metabolites) that drive resilience or susceptibility, hence, integrating environmental metadata (e.g., temperature, humidity, pathogen load) with molecular features enhance the production of models that are more applicable under field conditions.

### 9.3. Translational Steps: From Discovery to Crop Improvement

**Figure 5:**
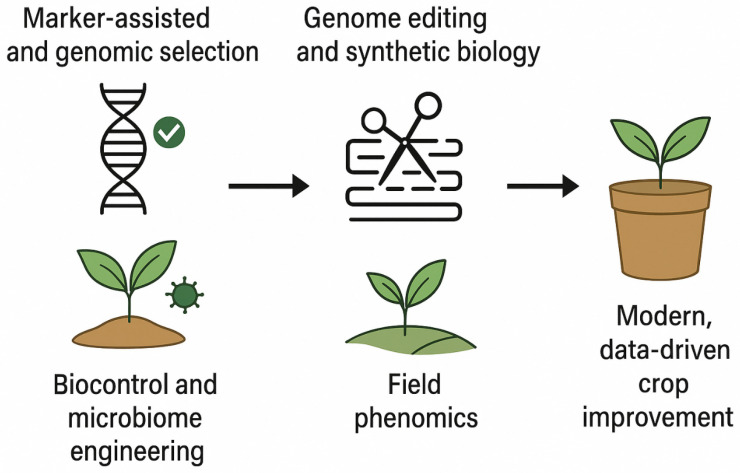
Key Translational Pathways Driving Modern, Data-Driven Crop Improvement.

The integration of multi-omics and gene regulatory network (GRN) analyses into practical breeding pipelines have represented a critical step toward achieving resilient and high-performing crops under the mounting pressures of climate change and global food insecurity. Translating systems-level insights into real-world agricultural innovations requires the convergence of advanced molecular tools, computational modeling, and field-based validation. As highlighted in Figure 5, the key translational pathways marker-assisted and genomic selection, genome editing and synthetic biology, biocontrol and microbiome engineering, and field phenomics that collectively form the foundation of modern, data-driven crop improvement.

Multi-omics and GRN studies have enabled the identification of hub genes and co-regulated modules that serve as key determinants of complex agronomic traits, including yield, stress tolerance, and disease resistance. These hubs or modules, once validated, can be developed into molecular markers for marker-assisted selection (MAS) or incorporated into genomic selection (GS) frameworks [78]. The use of GRN-derived markers provides a more holistic understanding of trait inheritance by accounting for epistatic interactions and regulatory hierarchies. For example, transcriptomic and proteomic markers associated with drought-induced regulatory modules have improved selection efficiency in crops such as rice and maize [58]. By leveraging omics-informed markers, breeders can shorten selection cycles and enhance prediction accuracy for complex traits, advancing precision breeding strategies that integrate molecular and phenotypic data.

Genome editing technologies, particularly CRISPR-Cas systems, offer direct avenues for manipulating key regulatory nodes identified through GRN analyses. By precisely targeting transcription factors, kinases, or receptor-like proteins, researchers can modulate entire defense or stress-response pathways to achieve enhanced resilience [66]. For instance, editing WRKY, NAC, and DREB transcription factors has been shown to improve tolerance to abiotic stresses such as drought, salinity, and temperature extremes. Synthetic biology complements this approach by allowing for the construction of synthetic promoters, regulatory circuits, and modular gene networks that fine-tune gene expression in response to environmental cues. Such systems-guided crop design enables the dynamic regulation of defense mechanisms without compromising growth or yield potential.

Recent advances in multi-omics have revealed the critical role of plant-associated microbiomes in maintaining health, productivity, and disease resistance. Multi-omics-guided microbiome engineering can identify beneficial microbial taxa, signaling molecules, and metabolic interactions that enhance host immunity and stress tolerance [79]. By characterizing root and phyllosphere microbiota using metagenomics, metatranscriptomics, and metabolomics, researchers can develop biocontrol agents that outcompete pathogens or prime host defense pathways. For example, beneficial rhizobacteria that trigger induced systemic resistance (ISR) have been successfully identified and applied to cereals and legumes through omics-guided screening. Integrating microbiome insights into breeding and management strategies represents a sustainable approach to reducing chemical inputs and improving ecological resilience in agroecosystems.

While omics and GRN studies provide molecular predictions of stress adaptation, field phenomics bridges the gap between laboratory discovery and real-world performance. High-throughput phenotyping platforms including drone imaging, hyperspectral sensors, and automated ground-based systems enable large-scale monitoring of physiological and morphological traits linked to omics biomarkers [80]. When integrated with transcriptomic, proteomic, and metabolomic data, phenomics allows for real-time validation of molecular predictions under dynamic environmental conditions. This convergence of multi-omics and phenomics facilitates the accurate identification of high-performing genotypes and supports the development of predictive models for crop performance across diverse agroecological zones.

In summary, translating multi-omics and GRN insights into crop improvement requires an integrative pipeline that connects molecular discovery, computational modeling, and field validation. Marker-assisted selection, genome editing, microbiome engineering, and field phenomics together constitute a powerful framework for designing next-generation crops capable of thriving under multiple stressors while sustaining yield and quality.

## 10. Ethical and Data-Sharing Considerations in Multi-Omics Plant–Pathogen Research

The growth of multi-omics research has underscored the need for ethical data governance, especially regarding open data, intellectual property, and benefit-sharing in global collaborations [70]. FAIR data principles (Findable, Accessible, Interoperable, and Reusable) have also remained essential in ensuring reproducibility and equitable access [73]. The fact that developing countries face challenges in computational infrastructure and bioinformatics training, initiatives such as the African BioGenome Project need to be strengthened further to promote inclusive capacity building, to ensure that genomic and omics advances benefit diverse agricultural systems [67]. There is also need to integrate local germplasm data with global repositories, while respecting national data sovereignty, an initiative that will support responsible innovation and enhance resilience research in underrepresented regions [51].

## 11. Integration Challenges, Future Directions, and Conclusion

### 11.1. Current Challenges in Multi-Omics Integration

Despite rapid advances in omics technologies, integrating multi-dimensional datasets remains a major challenge in systems-level plant biology. The foremost issue lies in the heterogeneity of data types, which differ in scale, noise level, and temporal resolution [45]. Transcriptomic, proteomic, and metabolomic data often exhibit weak correlations due to post-transcriptional regulation, metabolite turnover, and environmental variation [53]. Moreover, plant–pathogen and multi-stress systems exhibit strong context dependency, where responses differ across genotypes, developmental stages, and stress intensities [7,12]. Additionally, data standardization and interoperability present further obstacles. Although repositories such as NCBI GEO, PRIDE, and MetaboLights host large omics datasets, inconsistent metadata annotation and variable preprocessing protocols hinder cross-study comparison [30,34]. Additionally, missing data and batch effects compromise downstream integration, emphasizing the need for robust normalization and imputation methods [67].

From a computational standpoint, it has been demonstrated that integrating multi-layered omics requires scalable frameworks capable of handling non-linear relationships and high-dimensional features. While network-based integration and machine learning have improved predictive accuracy, model interpretability has remained limited [51,53]. The lack of standardized pipelines for multi-omics data fusion, visualization, and validation across species constrains reproducibility and cross-laboratory collaboration [51]. Moreover, biological validation of integrative models often lags behind computational advances. Specifically, experimental verification of predicted regulatory interactions, protein complexes, or metabolic fluxes being resource-intensive, requires multi-disciplinary expertise [52]. Bridging this computational experimental divide is one of the critical approaches that is necessary in translating omics insights into tangible agricultural outcomes.

### 11.2. Emerging Technologies and Integrative Strategies

Several emerging technologies are poised to overcome these integration barriers. Single-cell multi-omics which simultaneously profiles transcriptomes, epigenomes, and proteomes at cellular resolution has so far opened new avenues for understanding spatial and temporal heterogeneity in plant stress responses [28,32]. Techniques such as spatial transcriptomics and imaging mass spectrometry are being used to reveal cell-specific signaling gradients within infection sites or stress-affected tissues [12]. Artificial intelligence (AI) and deep learning approaches are also increasingly being used to predict gene regulatory interactions and network topology. Graph neural networks (GNNs) and autoencoders have been used to integrate heterogeneous omics data and infer hidden dependencies that govern stress adaptation [58,61]. Therefore, coupled with causal inference and dynamic Bayesian models, these frameworks have continued to enable predictive simulation of stress responses, thereby accelerating gene discovery for resilience breeding [22,26].

The integration of multi-omics with phenomics, that include high-throughput imaging and remote sensing represents a powerful translational shift. Phenomics has so far supported the linking of molecular-level changes to physiological performance under field conditions, bridging the genotype–phenotype gap [57]. By combining omics-derived biomarkers with sensor-based phenotyping, researchers have managed to model real-time stress dynamics and identify elite cultivars with superior tolerance profiles [62]. On the other hand, synthetic biology has also played a transformative role in functional validation. Indeed, CRISPR/Cas-based multiplex editing allows for precise manipulation of GRN hubs and feedback circuits identified through omics integration [60]. These systems have been engineered to fine-tune hormonal cross-talk, antioxidant defense, and secondary metabolism pathways, enabling the rational design of multi-stress-resilient crops [67].

### 11.3. Ethical, Data-Sharing, and Capacity Considerations

As multi-omics research expands, ethical considerations surrounding data ownership, access, and benefit-sharing need to be critically addressed, especially in biodiversity-rich regions such as Africa and Southeast Asia. Open-access repositories and FAIR (Findable, Accessible, Interoperable, and Reusable) data principles are essential for equitable scientific progress [75]. Moreover, capacity building in bioinformatics and systems biology is vital for developing countries to fully benefit from omics technologies. Collaborative networks such as the African BioGenome Project and Plant Genome Integrative Platforms aim to strengthen analytical infrastructure and promote data democratization [71]. Integrating local knowledge systems with global data frameworks will ensure context-relevant application of multi-omics in sustainable agriculture and food security.

### 11.4. Future Directions

Future omics research should emphasize dynamic, integrative modeling that captures temporal and spatial complexity across stress combinations. Multi-modal integration of transcriptomics, proteomics, metabolomics, and epigenomics should be paired with fluxomics and phenomics to reconstruct whole-plant system behavior [51]. Predictive network modeling should move beyond correlation-based inference toward causal frameworks that explain regulatory hierarchies and signal propagation. Such models can identify “master regulators” and “network motifs” that underlie robustness or fragility in plant stress networks [72]. Moreover, translational pipelines linking omics discovery to breeding programs should be institutionalized. Integrative databases and genome-to-phenome portals could accelerate marker-assisted and genomic selection for multi-stress tolerance [45]. Partnerships between academia, industry, and policy institutions will be essential for scaling these approaches to global agricultural systems.

## 12. Conclusions

Integrative multi-omics and gene regulatory network (GRN) approaches are transforming our understanding of plant–pathogen interactions by highlighting the multilayered transcriptional, post-transcriptional, and post-translational regulatory architectures that shape immunity; however, fully realizing this potential requires overcoming persistent challenges in cross-platform normalization, data harmonization, and the integration of heterogeneous datasets across spatial, temporal, and cellular scales. Moving forward, explicit targets for improving data integration in plant immunity include constructing high-resolution, context-specific GRNs that capture dynamic interactions among key molecular actors, like pattern-recognition receptors (PRRs), mitogen-activated protein kinase (MAPK) cascades, nucleotide-binding leucine-rich repeat receptors (NLRs), transcription factors like WRKYs and NACs, and immune-regulating small RNAs—while ensuring that proteomic layers (ubiquitinomes and phosphoproteomes,) are linked seamlessly with metabolomic and transcriptomic outputs. For networking purposes, standardized frameworks for multi-omics network reconstruction, causal inference, and cross-study comparability will be essential to uncover conserved and divergent immunity modules across species. Plant immunity is governed by multilayered defense mechanisms, including pattern-triggered immunity (PTI) and effector-triggered immunity (ETI), both driven by rapid transcriptional and proteomic reprogramming. Core processes such as receptor-mediated pathogen perception, MAPK signaling, hormone-regulated defense pathways, ROS bursts, calcium signaling, and activation of pathogenesis-related proteins form the molecular foundation of these responses, and we highlight how transcriptomic and proteomic datasets effectively capture early signaling dynamics, post-translational modifications, and defense metabolite accumulation. To better balance this mechanistic overview with methodological advances, the revised manuscript incorporates updates on cutting-edge transcriptomic tools, including single-cell and spatial transcriptomics and full-length Iso-Seq, alongside recent proteomic innovations such as DIA-MS, phosphoproteomics, and PTM-focused workflows.

We also emphasize experimental and analytical considerations central to plant immunity studies, including the need for precise time-course sampling, adequate biological replication, consistent pathogen inoculation, and normalization strategies that address batch effects, infection-induced expression spikes, and compositional changes. Furthermore, challenges in cross-omics integration—such as dynamic-range differences, missing values, and transcript–protein timing mismatches—are discussed. Finally, while networking approaches remain valuable, the Perspectives Section now incorporates complementary strategies including metabolic pathway analysis, merged gene–protein–metabolite networks, dynamic metabolic modeling, and multi-layer fusion frameworks that broaden analytical possibilities and address current limitations in plant immunity research. Advances in single-cell and spatial omics, AI-driven network learning, and synthetic biology provide promising avenues to refine these integrative models and enable predictive, design-based manipulation of immune pathways [62,69,71]. Coupled with open data infrastructures, ethical oversight, and interdisciplinary capacity building, these innovations will allow multi-omics not only to decode the complexity of plant defense networks but also to generate actionable targets for engineering climate-resilient, disease-resistant crops that support global food security.

## Figures and Tables

**Table 2:** Summary of integrative multi-omics insights into plant–pathogen interactions across major model and crop systems.

Pathosystem	Omics Layers Integrated	Key Insights	References
*Arabidopsis–P. syringae*	Transcriptome + Proteome	Identification of transcriptional hubs WRKY70 and NPR1	[53]
*Rice–M. oryzae*	Transcriptome + Metabolome	Hormone–metabolite cross-regulation in SA and JA pathways	[56]
*Wheat–F. graminearum*	Proteome + Phosphoproteome	Signaling cascades underlying cell wall fortification	[30]
*Tomato–Botrytis cinerea*	Transcriptome + Proteome + Interactome	Multi-layer regulation of oxidative stress tolerance	[77]

## Data Availability

No new data were created or analyzed in this study.
